# Populist radical right parties and discursive opportunities during Covid-19. Blame attribution in times of crisis

**DOI:** 10.1007/s12286-022-00540-w

**Published:** 2022-11-21

**Authors:** Jakob Schwörer, Belén Fernández-García

**Affiliations:** 1 Institute for Political Science, Leuphana University, Lüneburg, Germany; 2 Department of Political Science, Public International Law and Procedural Law, Faculty of Law, University of Málaga, Málaga, Spain

**Keywords:** Covid-19, Radical right, Populism, Nativism, Content analysis, Social media, Covid-19, Radikale Rechte, Populismus, Nativismus, Inhaltsanalyse, Soziale Medien

## Abstract

This study examines how populist radical right parties (PRRP) adapt to the discursive opportunities delivered by the Covid-19 pandemic. Conducting manual content analyses of discourses on Twitter in six Western European countries between February and October 2020 we show that PRRP frame the pandemic as a domestic political crisis attacking primarily national political elites. While PRRP occasionally link their initial support for measures against the pandemic to blame attribution towards immigrants accusing them of spreading the virus, nativist messages almost disappear when PRRP became less supportive of restrictions. Instead, in countries less affected by the pandemic (Austria, Germany) as well as in Spain, PRRP compensate the lack of nativist messages by using anti-elitist demonizing discourses against the national government accusing it of abolishing democracy and undermining freedom. The study shows how PRRP electorally “survive” periods with scarce nativist discursive opportunities by emphasizing on anti-elitist discourses.

**Supplementary Information:**

The online version of this article (10.1007/s12286-022-00540-w) contains supplementary material, which is available to authorized users.

## Abstract (de)

Diese Arbeit untersucht, wie rechtspopulistische Parteien (PRRP) sich an die diskursiven Gelegenheiten während der Covid-19-Pandemie anpassen. Inhaltsanalysen von Twitter-Diskursen in sechs westeuropäischen Ländern zwischen Februar und Oktober 2020 zeigen, dass PRRP die Pandemie als innerpolitische Krise kommunizieren, die von nationalen politischen Eliten verursacht wurde. Während PRRP ihre anfängliche Unterstützung für Maßnahmen gegen die Pandemie gelegentlich mit Schuldzuweisungen an Migrant:Innen verknüpfen, denen vorgeworfen wird Treiber der Pandemie zu sein, verlieren nativistische Botschaften an Relevanz sobald PRRP die Maßnahmen nicht mehr aktiv unterstützen. In Spanien und in Ländern die weniger von der Pandemie betroffen waren (Österreich, Deutschland), kompensieren PRRP das Fehlen nativistischer Botschaften mit anti-elitären, dämonisierenden Diskursen gegen die nationale Regierung, die sie der Abschaffung der Demokratie beschuldigen. Der Artikel zeigt, wie PRRP durch die Betonung populistischer anti-elitärer Diskurse, Perioden mit geringen nativistischen diskursiven Gelegenheiten elektoral „überleben“ können.

## Introduction

Populist radical right parties (PRRP) have become relevant challengers in almost all western European countries within the last decades. PRRP adopted a winning formula that combines populism and nativism by toning down extremist stances and references to fascism (Rooduijn and Akkerman 2015). Their populist discourses portray the people in a positive and monolithic way and oppose it to corrupt and evil political, economic or other type of elites (Mudde 2004). Their nativist stances portray specific out-groups—immigrants, asylum seekers, Muslims, ethnical groups—as a threat for the native society. Moreover, those perceived as threatening the homogeneous nation state—international organizations, “lefties” or progressives—are blamed for mass migration, multiculturalism and for harming the native people. Hence, both populist and nativist parties strongly depend on blame attribution to others (Hameleers and Schmuck 2017).

In general, critical events such as the so-called “refugee crisis” or the European financial crisis offer discursive opportunities for PRRP (Caiani and Graziano 2019) since populist and nativist messages can be easily applied to situations where elites and cultural, national or religious groups are in the focus of debates. Yet, the current Covid-19 pandemic delivers very exceptional and challenging discursive opportunities for PRRP (Bobba and Hubé 2020b). While the amount of scholarship about PRRP’s behavior during the pandemic recently increased considerably, we still lack systematic empirical and comparative knowledge about targets and content of PRRP’s blame attribution since the outbreak of the virus in Europe.

We argue that nativist discursive opportunities are considerably restricted for PRRP during the pandemic. While anti-elitist blame attribution may still be linked to the executives’ handling of the pandemic, nativist messages may be more difficult to sell in the face of a national (and international) health emergency in which other issues than immigration are salient. We further assume that the content of blame attribution towards elites depends on the extent the pandemic affected the individual countries in terms of death and infection rates. While countries such as Spain and France suffered strongly during the first wave, death rates in Germany and Austria have been much lower. This fact might not only explain the emergence (and non-emergence) of anti-restriction movements in these countries but may also determine the types of allegations addressed towards the government. In this sense, we assess the salience of nativist and populist blame attribution for PRRP during the pandemic and the content of allegation towards the “enemies” of the nation by conducting manual content analyses over the first eight months of the pandemic from the official Twitter accounts of six western European PRRP.

The paper is structured as follows: We first address the main ideological and discursive traits of the populist radical right, namely populism and nativism. Subsequently, we formulate our hypotheses based on literature on crises and populism and on the debate about the core ideological features of PRRP. We analyze discourses of PRRP on Twitter during the Covid-19 pandemic in Germany, Austria, Switzerland, France, Spain and Portugal. Before presenting our empirical findings, the methodological approach is described. We conduct classical quantitative content analyses of Tweets on Twitter observing which actors are criticized and blamed by PRRP and what the content of the blame attribution is about.

The results show that the domestic political elite is indeed the main target of all PRRP of the sample during the pandemic. Nativist messages are less important as well as other targets like scientific, external or media elites. It is mostly during PRRPs’ initial support for restrictive measures that nativist messages are linked to pandemic-related issues blaming supposed non-native outgroups, immigration and the lack of border protection of causing or worsening the spread of the virus. Opposition to measures against the virus erased nativist elements but triggered demonizing accusations against political elites blamed for undermining democracy and freedom.

The findings contribute to the debate about the salience of populist and nativist ideology for the western European populist radical right. While we still argue that nativism should be considered the core ideological feature of PRRP, the later can also refrain from using respective discourses by emphasizing on populist rhetoric. Thus, nativism may not be as indispensable to these parties as it is commonly assumed (Akkerman 2017; Rydgren 2017). Although nativism is considered a flexible ideology (Schwörer and Fernández-García 2020), we argue that populism is the more flexible concept allowing PRRP to electorally survive the pandemic by targeting political elites compensating for the lack of nativist discourses.

## The populist radical right

PRRP are defined as both populist and nativist. Starting with the first element, populism is defined as “an ideology that considers society to be ultimately separated into two homogeneous and antagonistic groups, ‘the pure people’ versus ‘the corrupt elite’, and which argues that politics should be an expression of the volonté générale (general will) of the people” (Mudde 2004, p. 543). While the ideological nature of populism is sometimes debated (Aslanidis 2016), several scholars—and even critics of the ideational approach—agree that populist *communicative* elements exist which construct an antagonism between a moral people and a corrupt elite that does not act in the people’s interest (Aslanidis 2016; Hawkins 2009; Jagers and Walgrave 2007; Müller et al. 2017; Reinemann et al. 2017; Rooduijn et al. 2014). For the purpose of this study, we consider populism as a communicative feature or discourse that derives from a certain populist belief or thin-centered ideology.^1^ More specifically, we are mainly interested in anti-elitist discourses and blame attributions: “By highlighting the purity of the people and by referring to the establishment as culprit, populism is inherently about attributing blame to others” (Hameleers and Schmuck 2017, p. 2). Elites are accused of harming the people, the nation or the “heartland” (Taggart 2000). Yet, who or what is framed as the evil elite differs: it can be politicians or parties in general (Rooduijn et al. 2014), the government or the EU (Hameleers and Schmuck 2017; Jagers and Walgrave 2007) but also non-political actors such as economic, cultural and media as well as foreign elites (Fernández-García and Luengo 2018). Which elites are attacked by populists is at least partially determined by the populists’ host ideology. Left-wing populist, for example, tend to criticize economic elites, the profiteers of capitalism or the rich besides political elites (Katsambekis 2017).

Besides blaming elites, PRRP further exclude religious, ethnic or cultural groups from society. This is caused by their host ideology often named “nativism” (Mudde 2007) or “ethnic nationalism” (Rydgren 2017). Both concepts are defined in very similar terms and mostly refer to the same traits of PRRP. According to Mudde (2007, p. 19), nativism is an ideology “which holds that states should be inhabited exclusively by members of the native group (‘the nation’) and that non-native elements (persons and ideas) are fundamentally threatening to the homogenous nation-state.” Like populism, nativism constructs in- and out-group. Yet, unlike populism, the radical right separates society in a horizontal dimension. Talking about ethnic nationalism, Pauwels (2014, p. 25) provides a concept very close to nativism defining it as an “idea that the nation and the state should coincide and that non-national elements (persons and ideas) are fundamentally a threat for the homogeneous state.” These two categories, the nation and the state, define “who is and who is not ‘native’” (Mudde 2007, p. 64) in the radical right worldview. Based on their populist and nativist ideologies, Mudde (2007) provides a typology of PRRPs’ enemies (Table 1).

**Table 1:** Enemies of PRRP. Own elaboration based on Mudde (2007, p. 65)

		Nation	
		Within	Outside
**State**	*Within*	*The elite* Politicians; parties; governments; cultural elites (writers, artists, intellectuals, scientists etc.); economic elites (big businesses, banks, the upper class etc.); media and journalists	*Non-natives/outgroups* Immigrants; asylum seekers; “illegals”; ethnic, religious and national minorities (Muslims; Jews; Turks; Arabs; Syrians; Asians etc.)
	*Outside*	*Compatriots abroad* State representatives in other countries or international organizations (e.g., ambassadors; officials in the European Union etc.); members of the nation residing in other states	*Externals* Countries; inhabitants of other countries; foreign elites (“globalist elites”, Bill Gates; George Soros etc.); international organizations (UN; EU; World bank etc.)

Those within both the nation and the state are national political, economic and cultural elites, demonized in both nativist and populist terms “as traitors to the nation” (p. 65) and accused of following a globalist agenda (i.e. mass immigration). Second, those outside of the nation but within the state consist of immigrants, asylum seekers as well as ethnic, religious and national minorities. Third, compatriots abroad (within the nation but outside the state), such as representatives of the state in supranational organizations, (e.g., officials or representatives in the European Union) are also attacked for not representing the interests of the native-people. Last, those outside both the nation and the state—external enemies—are believed to conspire against the nation. This type of enemy includes international organizations and foreign elites such as Bill Gates accused of planning a “vaccination dictatorship” during the pandemic (Höhn 2020). Therefore, we find a great variety of enemies in the discourses of the PPRR that respond to both nativist and populist reasons and, on many occasions, to a combination of both.

Within the last years, a debate about the importance of nativism and populism has emerged in academia. While nativism (or ethnic nationalism) is considered as the core ideological and discursive feature of PRRP (Akkerman 2017; Mudde 2007; Rydgren 2017; Schwörer 2021a), some scholars like Rydgren even question the use of the term populism for PRRP. In his view “it is misleading to label these parties ‘populist parties’ since populism is not the most pertinent feature of this party family” (Rydgren 2017, p. 486). In his logic, the exclusion of other cultures and supposed non-native ideas constitute the worldview of these actors and explain blame attribution towards elites accused of following a multicultural agenda. Akkerman (2017) argues in a similar vein stating that it is the “xenophobic and nationalist ideology” that characterizes PRRP rather than populism. Following these arguments and given the scarce nativist discursive opportunities provided by the pandemic (see sections below), PRRP should struggle finding other narratives in order to electorally survive the pandemic.

Finally, it should be noted that besides populism and nativism, authoritarianism is considered the third ideological element of PRRP, considered as “belief in a strictly ordered society, in which infringements of authority are to be punished severely” (Mudde 2007, p. 23). Authoritarianism seems to be activated in contexts that produce fear, threat and uncertainty such as the current health crisis, and might explain certain demands for strict measures against the spread of the virus and for sever consequences for those who do not comply. Although authoritarianism does not directly determine the targets of blame attribution, it is considered as one of the predictors of prejudice and intolerance. In the context of the pandemic, the study of Hartman et al. (2021) concludes that anxiety about the COVID-19 pandemic may trigger authoritarian attitudes on nationalist and anti-immigrant attitudes.

## The populist radical right, crises and discursive opportunities in times of Covid-19

Broadly speaking, the rise of populist and radical right parties is linked to structural social and cultural changes (Chiaramonte and Emanuele 2015; Norris and Inglehart 2019). Besides these *constantly* ongoing processes of change, crises as a “temporary worsening of the situation”^2^ (Spier 2010, p. 54) further provide opportunities for populist and radical right parties by bringing their issues and dominant discourses into public focus. That a “crisis of representation” (Bobba and Hubé 2020a) facilitate populist mobilization is common sense among academics (Canovan 1999; Kriesi 2015; Mouffe 2005; Spier 2010). Ernesto Laclau (2005) even claimed that populist parties cannot emerge without a political crisis while others deny that a crisis is necessarily a prerequisite for populists’ successes (Bobba and Hubé 2020a).

The recent rise of populist and nativist parties in Southern (M5S, Podemos, Syriza, Vox) and Central Europe (AfD, FPÖ) can be interpreted as a result of beneficial opportunities provided by political, economic and “cultural” crises populist parties have taken advantage on: “Broadly speaking, the crises are ‘windows of opportunity’ that can be used by the new political parties or for the rebranding of old political parties, which find it relatively easy to build a ‘thin-centered ideology’, especially in the context of the very widespread social media that further facilitates simplified, unilateral, and antagonistic forms of communication” (Caiani and Graziano 2019, p. 1150). Immigration for example, became a highly relevant topic in Germany during and after the so-called “refugee crisis” in 2015. The AfD has managed to further politicize this issue developing from a rather irrelevant right-wing niche party to the (temporarily) third-largest political actor of the country—in terms of votes. A more recent example is found in the territorial crisis in Spain, which has opened a window of opportunity for the radical right to mobilize centralist nationalist sentiments in the country (Turnbull-Dugarte 2019).

However, while we know how populist and radical right parties benefit from crises that contribute to the public salience and visibility of their core discourses and issues (i.e., immigration, national identity), we know little about how PRRP behave when certain events do not necessarily bolster discursive opportunities. According to Koopmans and Muis (2009, p. 648), “discursive opportunities” are “the aspects of the public discourse that determine a message’s chance of success in the public sphere.” The authors use this concept to analyze the likelihood of success for right-wing extremist mobilization, focusing on the visibility, legitimacy, and resonance that radical right-wing actors and their claims have in public discourse (Giugni et al. 2005; Koopmans and Muis 2009). Building on this approach, we argue that a favorable discursive opportunity exists when the core issues of PRRP coincide with the dominant issues in the public debate.

In this regard, the Corona pandemic can be considered as an “event” that highlights issues outside of the usual political domains political competition usually takes place, at least until summer 2020 (Bobba and Hubé 2020b). Besides “rally-’round-the-flag” effects where people unify in the face of common threats behind the executive (Murray 2017)—reflected for example in the increased vote share for German and Austrian center-right governing parties during the first months of the pandemic and temporary vote losses for Spanish, German and Austrian PRRP^3^—populist parties found it hard to make use of their classical tactics. The outbreak of Covid-19 and respective media coverage increases insecurity within societies and thereby shift the focus on existential issues and healthcare policy (Garfin et al. 2020) that have little to do with right-wing populists’ nativist discourses. According to Bobba and Hubé (2020b), the crisis has affected every aspect of private and public life reducing opportunities for populist and nationalist rhetoric. Especially if we consider nativist messages as crucial feature (Akkerman 2017; Rydgren 2017) and hence, indispensable for PRRP, discursive opportunities are severely restricted by the pandemic. It is true that the pandemic offers specific opportunities for nativist blame attribution towards migrant communities. Certain ethnic minorities or immigrant communities have indeed been attacked for putting the country’s public health at risk by spreading the virus. For example, Chinese people have been accused of spreading the virus, as well as immigrants and occasionally the Roma population (Baião 2020; Bobba and Hubé 2020b; Wondreys and Mudde 2020). Hence, in the context of the pandemic, immigrants can primarily be blamed for spreading the virus and for not following the recommendations and rules to decrease the spread. But even this discursive opportunity seems restricted by the fact that several PRRP quickly became opponents of restrictions and sometimes even denied the danger of the virus (Bobba and Hubé 2020b; Fiedler 2020).

Regarding blame attribution towards domestic and external elites, opportunities may lay a bit different. The political establishment, external or scientific elites can be blamed for both undermining freedom by implementing (or recommending) measures against the spread of the virus and for not reacting fast and strict enough to the outbreak of the pandemic. The spread of theories about conspiracies of political, cultural and economic elites aiming to suppress the European populations dictatorially through pandemic measures after the initial “shock” of the outbreak of the virus, may provide particularly promising opportunities for anti-elitist discourses during certain stages of the pandemic.

It is true that crises should not only be understood as exogenous events to which PRRP are passively exposed. According to Moffit (2015, p. 195), “populist actors actively perform and perpetuate a sense of crisis, rather than simply reacting to external crisis.” PRRP participate in what Moffit calls the “spectacularization of failure” underlying the crisis (p. 190). This allows populist actors “to pit ‘the people’ against a dangerous other, to radically simplify the terms and terrain of political debate and advocate strong leadership and quick political action to stave off or solve the impending crisis” (Moffit 2015, p. 190). Yet, neither Moffit would negate that the selection of the “dangerous other” also depend on external factors like the type of crisis and discursive opportunities. As mentioned above, we argue that it is primarily the political establishment together with external elites and scientists whose supposed failure is “spectacularized” rather than supposed non-native outgroups’ behaviour.

## Hypotheses

As described above, nativism (or ethnic nationalism) is often labelled the core feature of PRRP and anti-immigration and anti-outgroup messages constitute the core discursive elements of this party family (Mudde 2007; Rydgren 2017; Schwörer 2021b). Data from Schwörer (2021b) suggests that nativist messages against outgroups and immigrants tend to be more important for PRRP (before the pandemic) than blame attribution towards political elites (single parties/politicians; the government or the “political establishment” in general). We therefore expect that nativist messages against supposed non-natives and immigration in general do not simply disappear during the pandemic but that they play a less important role compared to populist anti-elitist discourses.

While framing immigrants as a threat for national health might be an option for PRRP which speak out in favour of measures against the virus, blaming the government and political elites of abolishing freedom by introducing restrictions might be a simpler and more promising type of blaming. The anti-lockdown protests in many European countries starting in spring 2020 may have offered further discursive opportunities for anti-elitist messages since several PRRP became opponents of restrictions and supportive of the protests (Bobba and Hubé 2020b; Fiedler 2020). We therefore expect that anti-elitist messages towards domestic political elites can be found more frequently during the pandemic than discourses against supposed non-native outgroups and immigration.

### H1

Discourses against domestic political elites (the government/other parties) are more widespread than nativist messages against “outgroups” and immigration.

We further assume that non-political elites “within the country and the state” play a certain role in PRRP’s blame attribution during the pandemic. The critical stance towards pandemic-related measures of parties like the German AfD also resulted in scepticism towards scientific experts and academic consensus (Wondreys and Mudde 2020). We find very similar patterns in other political domains like measures against climate change framed by PRRP as an agenda by an “illegitimate liberal, cosmopolitan elite” to suppress the people (Lockwood 2018, p. 726). While academics and scientific experts are usually no dominant target of PRRP blame attribution (Schwörer 2021a; Luengo and Fernández-García 2018), this may be different during the pandemic. Politicians strongly rely on scientific expertise which put virologists and experts from related fields in the focus of politics and the media (Lavazza and Farina 2020). We therefore assume that scientists are at least occasionally attacked by PRRP.

### H2

Scientists are target of blame attribution during the pandemic.

**Fig. 1:**
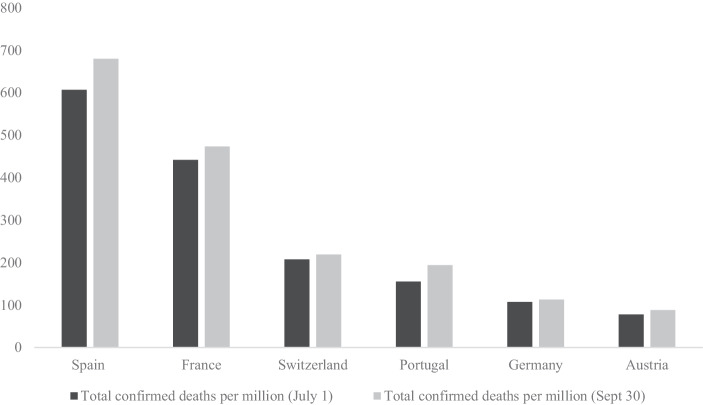
Total confirmed deaths per million (until July 1/September 30). (Source: data from https://ourworldindata.org/.)

Besides the question about the targets of blame attribution, the content of these discourses may vary between countries. One might assume that in countries which had been hit hard by the first wave in terms of death rate—Spain and France—PRRP may be less hostile towards the measures of the government than in countries which were not affected to the same extent like Germany and Austria (see Fig. 1). Political observers already emphasized that especially in the German and to a lower extent Austrian context, anti-restriction movement—often influenced by conspiracy theories—grew since spring 2020 while the same was not observed to the same extent in southern European countries (France24 2021). In this regard, PRRP in the countries hardest hit by the first wave of the coronavirus may criticize the late and inappropriate response of the government, demanding additional measures or at least the maintenance of existing ones while PRRP in the least affected countries may primarily demand less restrictions blaming the government of abolishing freedom.

### H3

PRRP in countries with lower death tolls after the first wave demand fewer restrictions from the government than PRRP in countries with higher death rates.

However, different types of accusations towards the government may further have a temporal dimension. Political observers already mentioned that several PRRP first supported the government in their handling of the pandemic (“rally around the flag” effect) while becoming anti-lockdown parties after initial support for the government (Bobba and Hubé 2020b; Lembcke 2020). In this regard we expect that PRRP were more in favour of the governmental agenda during the first wave while rejecting the measures of the executive in the aftermath accusing it of restricting constitutional rights and freedom.

### H4

Support for restrictions by PRRP towards the government follows a declining time trend.

## Research design and method

The study focuses on Western European countries where PRRP constitute electorally successful challengers. Furthermore, we are interested in the first reaction of PRRP to the pandemic and the following development of their discourses. Since political observers usually argue that PRRPs’ positions changed after the first weeks of the pandemic, our period of investigation needs to start at its beginning (Bobba and Hubé 2020b). Moreover, while some countries strongly suffered under the pandemic others have luckily been less affected during the first wave. The different levels of concern might also have an impact on the populist radical right and its communication. Therefore, we select countries where infection and death rates were particular high and where Covid-19 overburdened national health systems as well as states that were able to avoid a collapse of the health care systems and where infection and death rates have been rather low (as of September 2020). In sum, we analyze PRRPs’ discourses in Germany, Austria, Switzerland, Spain, France and Portugal during the outbreak of Covid-19. The selection of parties includes the Freedom Party of Austria, the Swiss People’s Party, the French National Rally, the Alternative for Germany, the Spanish Vox and the Portuguese Chega. All these parties are classified as PRRP in the literature (Rooduijn et al. 2019). We start our period on February 15 and terminate the analysis September 30 in 2020. It is particularly the first wave where governments’ restrictions have been particularly severe and where PRRP had to adapt to a new discursive scenario. In all of the countries under analysis, measures to prevent the spread of the virus began at the beginning or in mid-March 2020.^4^ Starting in mid-February allows to capture the first responses of PRRP to the pandemic situation. Ending our period of analysis at the end of September allows to analyze the discursive developments after the first lockdowns in these countries. The suggested period covers both, periods with harsh measures (“lockdowns”) and the following period with a lower spread of infections and less state interventions. It is particularly the second period political observers expect shifts in the positions of PRRP in the face of a decreased spread of infections (Bobba and Hubé 2020b).

We select Tweets on Twitter of official party accounts for measures of blame attributions. Twitter and social media in general can be considered as one of the most important arenas for populist campaigning (Ernst et al. 2019). Tweets of parties respond to current political issues what further allows analyses over time. Unlike party leaders, the statements of the official party account can be considered more representative for a party. We collect the Tweets via the rtweet package. Table 2 shows the selected parties, their Twitter profile and the number of analyzed Tweets.

**Table 2:** Case selection and Twitter profiles

Populist radical right party	Twitter profile	*n* Tweets
Alternative for Germany (AfD)	@AfD	1547 (256)
Freedom Party of Austria (FPÖ)	@FPOE_TV	789 (56)
Swiss People’s Party (SVP)	@SVPch	228 (56)
National Rally (RN)	@RNational_off	1920 (599)
VOX	@vox_es	6290 (1311)
Chega	@PartidoCHEGA	601 (78)

Number of Tweets shows the whole number published between 15/2 and 1/10/2020. Numbers in brackets show the number of pandemic-related Tweets

Methodologically we select a partially computer-based quantitative content analysis (Rooduijn and Pauwels 2011; Schwörer and Fernández-García 2021). We created a dictionary of keywords referring to the Covid-19 pandemic based on theoretical reasoning and explorative pre-tests (available in the online appendix). After running the keyword search, we analyze every Tweet containing a keyword manually in order to trace the concrete meaning and target of the accusation or demand. Hence, we do not analyze all Tweets since the beginning of the pandemic but only those referring to the virus since we are interested in enemy constructions *related* to the pandemic. We first distinguished between different types of targets based on Mudde’s distinction between different types of elites and non-natives. The relevant targets at least occasionally criticized are political, media and economic elites (“elites within”), the European Union and other international organizations (“externals”) as well as non-native outgroups. We further created a nativism-score consisting of attacks towards supposed non-natives as well as negative evaluations of immigration as such (with or without mentions of concrete groups or individuals) and positive evaluations of national sovereignty and border protection. The latter mostly consists of demands for border controls and economic and medical sovereignty as a consequence of the pandemic.

In order to know how reliable our measurement of targets actually is, we calculated Cohen’s Kappa. Therefore, a random selection of the French Tweets coded by the first author were coded by the second author according to the same rules as well as the same number of Tweets (random sample) which contain a pandemic-related keyword but which were not coded according to the respective category. Table 3 shows that for most targets (except economic actors) Cohen’s Kappa lies above 0.8 and is statistically significant indicating that inter-coder reliability is almost perfectly consistent (Landis and Koch 1977).

**Table 3:** Inter-coder reliability score for identification of negative targets’ evaluation

Accusation towards	Cohen’s Kappa	*n* (Tweets)
Political elites	0.889***	18
Media elites	0.857***	14
Economic elites	0.714**	14
Non-natives	0.833**	12
European Union	1.00***	14

***p < 0.001, **p < 0.01, *p < 0.05

Besides tracing the targets of accusation, we identified different types of content. This was mainly done inductively. Regarding the major target—the political elite—we distinguish between accusations related to the management of the pandemic and other non-pandemic related attacks. The concrete categories as well as their operationalization and example sentences can be found in the appendix and are shortly explained in what follows:

- *General accusation of bad management without clear tendency*: Political elites (mostly the national government) are blamed for a bad crisis management in general. It is not stated whether the targets should follow a more restrictive or a laxer approach towards the spread of the virus.
- *Manipulation, spreading lies and fake news:* Political elites are accused of not telling the truth, of hiding certain pandemic-related information, of manipulating public opinion or causing panic. This can be both in the context of demands for stricter or softer measures towards the spread of the virus but is only coded if there is a direct link to the management of the pandemic.
- *Anti-democratic attitude:* Political elites are portrayed as anti-democratic and accused of not tolerating opposition to their management of the pandemic. This is only coded if the accusation refers to the management of the pandemic.
- *Demands for measures to fight the pandemic:* Political elites are accused of not taking the pandemic serious enough and of not taking sufficient measures to fight the pandemic (e.g., lockdowns, more testing, obligation to wear face masks, etc.). Also support of existing measures is coded.
- *Demands for less restrictions and measures*: Political elites are accused of taking the pandemic too serious and of taking too restrictive measures (e.g., lockdowns, testing, obligation to wear face masks, etc.).
- *Nationalist and nativist demands and accusations*: Demands for nationalist policies including measures and accusations against non-natives and for border protection. Also demands for a strong nation state and against solidarity with other countries are coded if they appear in a pandemic-related context (related to measures but also to economic/social aspects of the pandemic management).
- *Worsening and producing economic/social problems*: Political elites are accused of not providing sufficient help for economic sectors or vulnerable groups and of wasting public resources. This category also includes demands for not harming the economy and is linked to pandemic contexts (related to economic/social consequences of the pandemic management).
- *Other non-pandemic related accusation*: All kinds of negative evaluations of political elites not related to the pandemic management or economic and social consequences of the spread of the virus. E.g.: accusations of being corrupt, selfish, not interested in the will of the people if this critique is not justified with the pandemic management.

Accusations towards non-native outgroups—the second most frequently occurring category—differ from attacks towards political elites. We distinguish between two pandemic-related categories (contributing to the spread of the virus/do not accept restrictions; are preferentially treated during the pandemic) and all other types of accusations not related to their behavior during in the context of the pandemic (see appendix—also for further categories). We further created an overarching nativism score, which includes all negative evaluations of non-native outgroups as well as negative evaluations of immigration as such (also without naming concrete outgroups) and positive references to national sovereignty or the protection of border in the context of the pandemic.

## Analysis

We start this section with the salience of pandemic-related issues for populist radical right parties. The parties of the sample dedicate very different amounts of Tweets to pandemic-related topics. Table 4 illustrates the percentages of Tweets within the period of analysis that contain a pandemic-related keyword (“% keywords”). While some parties—such as RN, SVP and Vox—often talk about pandemic-related issues, others like FPÖ, Chega and AfD address the health crisis less frequently in their communication on Twitter. This suggests that some radical right parties are willing to engage with the new situation and the new party system agenda while others rather refuse to be discursively dominated by the pandemic. Table 4 further shows how many of the pandemic-related Tweets contain blame attributions towards elites or outgroups (“TOT blaming”). While PRRP usually attack other actors in about half of those Tweets, the Spanish Vox party provides an exception, using in almost 80% of its pandemic-related messages forms of blame attribution.

**Table 4:** Frequencies and shares of pandemic-related communication in Twitter

Party	TOT Tweets	TOT keywords	% keywords	TOT blaming
RN	1920	599	31.2	358 (59.77%)
SVP	228	56	24.56	30 (53.57%)
Vox	6290	1311	20.84	1030 (78.57%)
AfD	1547	256	16.55	149 (58.2%)
Chega	601	78	12.98	39 (50%)
FPÖ	789	56	7.1	27 (48.2%)

The different visibility given to the health crisis may be related to the severity of the pandemic during the first wave in each country. In this regard, the first wave was particularly harsh in France and Spain and less devastating in Germany and Austria while death rates in Switzerland and Portugal were slightly higher than in Austria and Germany but much lower compared to France and Spain (see Fig. 1). In this regard we can explain that the French RN and the Spanish Vox frequently refer to the pandemic and that the FPÖ hardly does. Yet, especially the fact that the SVP scores second highest cannot be fully explained given Switzerland’s “moderate” death toll compared to Spain and France, although compared to neighboring Austria, its death rate is almost tripled.

While our analysis emphasizes on communication in the context of the pandemic, it is worth to provide at least some insights into the content of non-pandemic related Tweets. A non-representative random sample of Tweets from the parties under analysis (one Tweet per month) shows that PRRP do “business as usual” addressing traditional topics that can be linked to nativist and populist communication (see online appendix). As it might be expected, PRRP parties emphasizes immigration, left-wing extremism, and insecurity among other issues, displaying anti-elitist and authoritarian attitudes. They also focus on more specific and national issues such as scandals affecting domestic political elites (e.g., the FPÖ with the Wirecard and Ibiza-scandal of the ÖVP), the nationalist tensions in the country (Vox), or show opposition to specific measures or policies (e.g., the RN’s opposition to Macron’s pension reform or AfD’s opposition to climate measures). They also use Twitter to promote public events of their parties and to share favorable opinion polls for the own party (e.g., Chega).

**Fig. 2:**
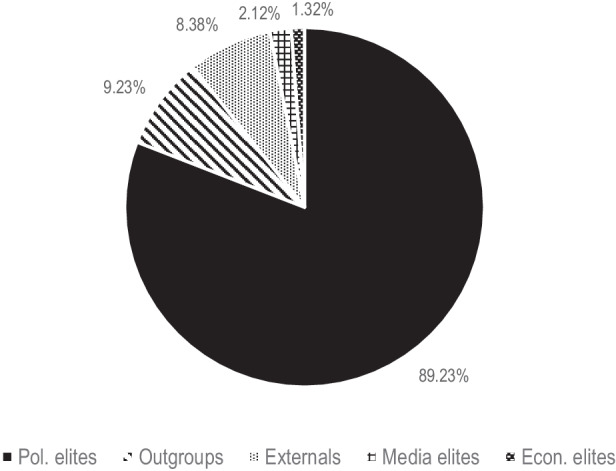
Average shares of targets attacked by PRRP (percentages). (Percentages of Tweets containing an attack towards specific targets on the total amount of Tweets containing a critique towards any kind of target. The sum of the percentages is more than 100 since Tweets can be assigned to multiple categories (e.g., attacks towards political elites and outgroups in the same message). SD: Political elites = 6.67, Outgroups = 6.18, Media elites = 3.43, Economic elites = 1.57, Externals = 7.23)

Starting with the targets framed as enemies, Hypothesis 1 assumes that political elites are more frequently addressed than alleged non-native outgroups, Fig. 2 illustrates the shares of specific targets on the whole number of Tweets attacking any type of actors within the period of analysis.^5^ The pie chart shows the mean scores of all parties of the sample. Political elites (“the elite within”) are most frequently criticized or confronted with demands by the populist radical right. Economic and media elites hardly play a role. Supposed non-native outgroups are indeed hardly criticized, unlike in non-pandemic times (Schwörer 2021b), what confirms Hypothesis 1. “Externals” are occasionally criticized as well—while “compatriots abroad” (outside the state but within the nation) are not addressed at all. Externals are mostly institutions and officials from the European Union but also other countries (i.e. China) or the WHO. In sum, the figure suggests that domestic political elites are the core targets of PRRP blame attribution during the pandemic. Hence, despite its global dimension, the crisis is mostly interpreted as a *national* event, managed badly by the *national* political elite. However, the considerable standard deviations indicate differences among the different parties of the sample. For example, it is almost only the AfD (6.52% of all Tweets targeting actors and 3.52% of all pandemic-related Tweets) and Vox in Spain (11.27% and 8.54% respectively), which target the national media, while SVP, FPÖ and Chega do not attack the media at all—and RN in France only four times in total. Political elites from the own country are the most often attacked targets among all PRRP—even though the frequency differs to some extent between the single parties.

Rather surprisingly, Hypothesis 2 cannot be confirmed. Scientists and academic elites are not portrayed in a negative light by most PRRP on Twitter until October 2020. Vox in Spain seems to be the only exception (six negative references to scientists), together with one Tweet from Chega. Vox attacks experts involved in certain media outlets and those who advise the government accusing them of having denied the seriousness of the pandemic at the beginning. Later Vox accuses experts of dramatizing the pandemic and exaggerating its consequences in order to impose the ideological agenda of the left and of having an ideological bias that seeks to favor left-wing parties (e.g., “The @rtve experts on coronavirus crisis [are] always with a social-podemite [pro Podemos] bias.”). In Chega’s case, one Tweet accuses specialists of hiding information about infections and deaths in order to protect the government.

**Fig. 3:**
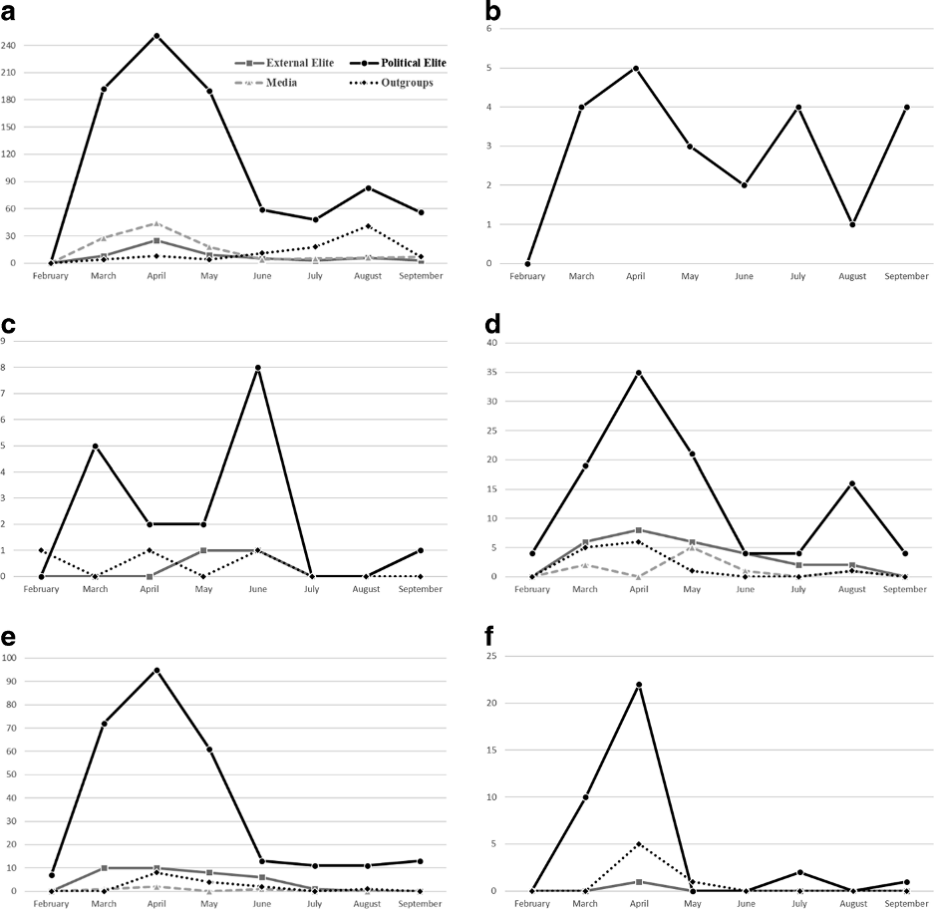
Evolution of enemies/targets of PRRP over time. **a** Vox, **b** FPÖ, **c** SVP, **d** AfD, **e** RN, **f** Chega

But can we observe an emphasis on specific targets during certain phases of the pandemic? First of all, Fig. 3 indicates that blame attributions are concentrated mainly in the first months of the pandemic, especially between March and June. The most frequent target, as previously mentioned, is the political elite concentrating most of PRRPs’ attacks in the six countries. Over the whole period of analysis, domestic political elites remain the main target of PRRP providing further support for H1. The peak of attacks on this target is reached in April in almost all cases (except SVP), coinciding with the hardest moment of the first wave. From then on, the attacks follow a decreasing trend in general terms—with slight exceptions among the SVP—to rise again slightly in August or September in some countries, when the second wave begins. The slightly different picture among the SVP may point towards its incumbent status. Populist parties in coalition governments are sometimes expected to decrease their anti-elitist rhetoric (Bernhard 2020), which is also reflected in the lowest share of messages against national political elites among the SVP (32% of pandemic-related tweets) compared to the other PRRP. The media and external elites also receive more attacks at the beginning of the pandemic than at the end of the first wave. The media are accused of lying and manipulating information related to the pandemic or of protecting national governments, while external elites are accused of not having prevented and acted in time (e.g., EU and WHO), of lying and spreading the virus (e.g., China), and of taking advantage of the pandemic imposing their globalist agenda (e.g., “the globalist elites”). As for outgroups, we can observe a general trend whereby attacks on these types of actors (mainly immigrants and refugees and in the case of Chega, also Roma people) occur at the beginning of the pandemic. Exceptions are the FPÖ, which does not attack any outgroup directly and—as mentioned below—the Spanish Vox, which follows a different time trend.

Diving a bit deeper into the distribution of nativist messages between the single PRRP, Table 5 shows the frequency of negative references to outgroups, to immigration (not necessarily including references to outgroups) and positive references to national sovereignty and border control. The overall nativism score reflects the share of Tweets containing at least one of these elements on the total amount of pandemic-related messages and illustrates the saliency of such discourses. The table suggests that there are considerable outliers regarding the quantity of nativist discourses. The Swiss SVP managed to link nativist elements to the pandemic in almost every fourth Tweet—equating 43% of all coded Tweets—while the FPÖ almost never links nativist messages to pandemic-related issues. This indicates that PRRP respond differently to the health crisis, which does not offer nativist discursive opportunities per se. The fact that the SVP tries to blame outgroups more often than the other parties may be related to its incumbent status: as a member of the government, it may shift blame attribution to actors outside the government.

**Table 5:** Frequency of nativist elements

	Overall nativism (in %)	Anti-outgroups (in %)	Anti-immigration (in %)	Pro sovereignty/border control (in %)
SVP	23.21 *(43.33)*	5.36 *(10.0)*	8.93 *(16.67)*	12.5 *(23.33)*
RN	11.85 *(19.83)*	2.5 *(4.2)*	2.5 *(4.2)*	8.01 *(13.41)*
Chega	11.54 *(23.08)*	7.69 *(15.39)*	6.41 *(12.82)*	3.85 *(7.69)*
Vox	10.45 *(13.30)*	7.09 *(9.03)*	8.01 *(10.19)*	2.06 *(2.62)*
AfD	9.38 *(16.12)*	5.08 *(8.73)*	1.56 *(2.69)*	3.52 *(6.04)*
FPÖ	1.79 *(3.7)*	0	0	1.79 *(3.70)*

Percentages of Tweets containing an attack towards specific targets on the total amount of *pandemic-related Tweets*. The scores in brackets show the percentages on the total amount of Tweets containing any type of coded element

**Fig. 4:**
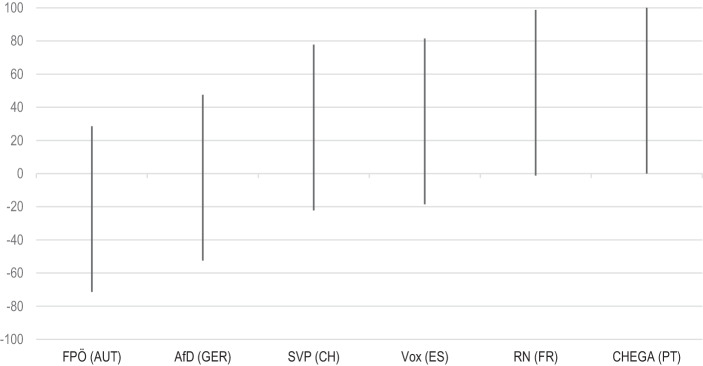
Share of demands for stricter/additional measures and for less restrictions. (Percentages of messages on the total amount of Tweets containing demands for or against measures towards elites)

**Fig. 5:**
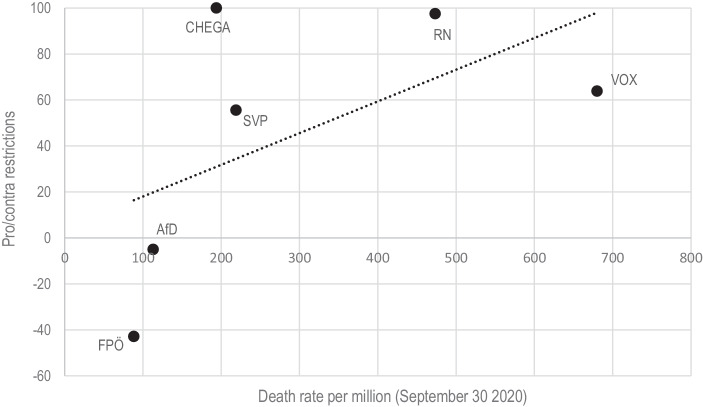
Net evaluation of measures and national death tolls. (Net percentages of measures’ evaluation (demands/stances for measures—demands against measures))

Moving on to the content of accusation towards political elites, it is expected that PRRP demand more or less measures to prevent the spread of the virus from the government depending on how affected the individual countries are by the pandemic (Hypothesis 3). As independent variable we select the official death toll of the countries and expect that PRRP evaluate measures to prevent the virus more often positive when the country was strongly affected by the pandemic. First, Fig. 4 illustrates the percentage of demands in favour of restrictions (positive values) and the percentage of demands for less restrictions (negative values) on the total amount of tweets containing a pro- or contra-demand regarding restrictions. It is only PRRP in Germany and Austria predominantly evaluating the measures negatively, indicating a preference to reduce restrictions. In line with H3 we further observe that Vox in Spain and RN in France—countries with the highest death rate as of the end of September 2020—primarily are in favour of governmental restrictions (or demand further measures). Fig. 5 illustrates the net evaluation index of measures (demands for measures—demands against measures) and the death rates on the x‑axis. We mostly can confirm our hypothesis with the exception of Chega in Portugal. While the country was less affected by the pandemic than Spain and France, the party always remained in favor of restrictions. Thus, while it seems reasonable that the different degrees of devastation the pandemic has caused in the single countries influences communication strategies of PRRP, it is not the only explanation.

**Fig. 6:**
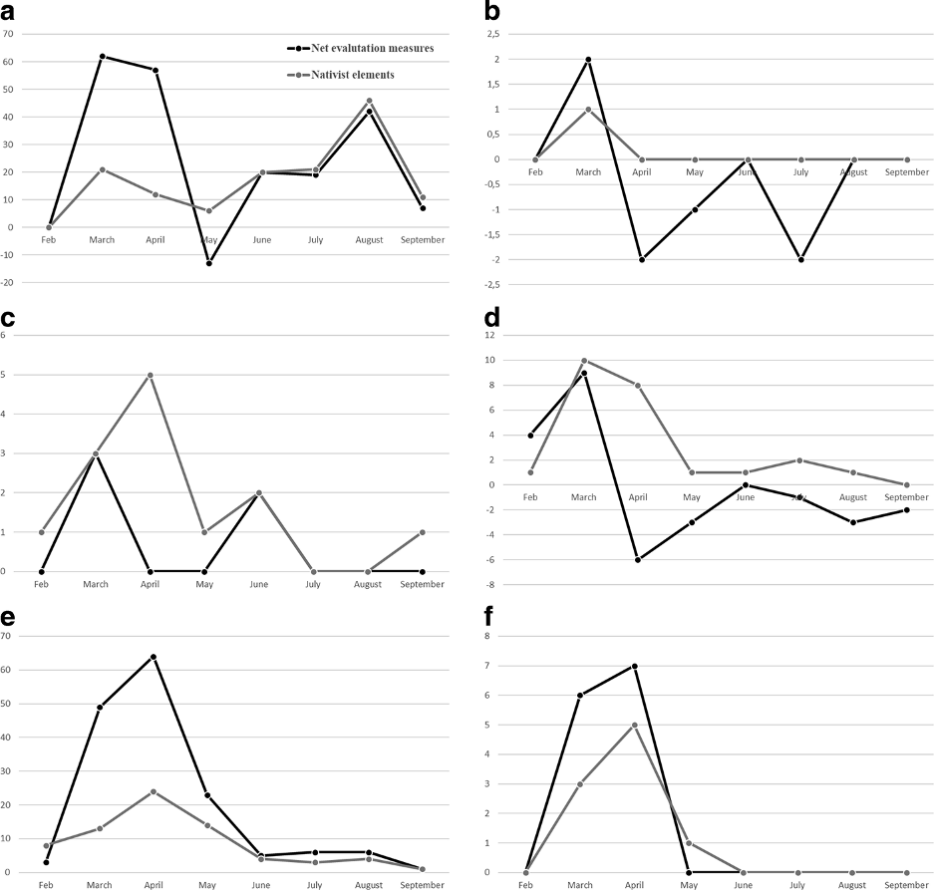
Net evaluation of governments’ measures against the pandemic and nativist elements. **a** Vox, **b** FPÖ, **c** SVP, **d** AfD, **e** RN, **f** Chega. (Positive values indicate support for measures against the pandemic while negative values indicate demands for less strict measures)

Hypothesis 4 assumes that the evaluation of governments’ measures further shifts over time in a more sceptical direction. At this point we come back to the role nativist messages play in the pandemic. Fig. 6 shows the net evaluation of governments’ measures over time. Negative values indicate a critical stance towards existing measures while positive values indicate demands for maintaining restriction or for additional ones. First, we can confirm H4: PRRP in all countries raise demands for measures towards the government at the beginning of the pandemic while they decrease over time or even turned into anti-restriction demands (AfD; FPÖ).

The figure also shows the frequency of nativist messages during the period of analysis.^6^ Nativist elements can mostly be observed during the first months of the pandemic until May 2020 among all parties of the sample except Vox. This coincides with the evaluation of governments’ measures: after accusing parties and politicians—mostly the national government—of not taking the crisis seriously and of not acting strict enough to the outbreak of the virus at the beginning of the pandemic, most PRRP change their strategy or at least hardly emphasize the risks of the pandemic and the need of severe measures after May. While demands for lockdowns and restrictions have been used to blame immigrants and outgroups to contribute to the spread of the virus and to demand the closure of the national borders, this communication strategy lapses when the pandemic is not considered a serious problem anymore. In this sense, nativist messages strongly depend on demands for measures to fight the pandemic. Vox’s exception to this pattern is explained by a contextual factor in Spain: the number of immigrants arriving in Spain by sea rises considerably during the summer months, which is reflected in the evolution of Vox’s discourse against immigration and outgroups (mostly, “illegal immigrants infected by Covid-19”). Like the rest of the PRRP, Vox’s accusations towards the government of not taking enough measures or of worsening the spread of the virus is related to the attribution of blame to immigrants, immigration and the lack of border control, especially in the summer months. Thus, the difference occurs mainly in temporal terms due to the specific migratory context of Spain.

**Fig. 7:**
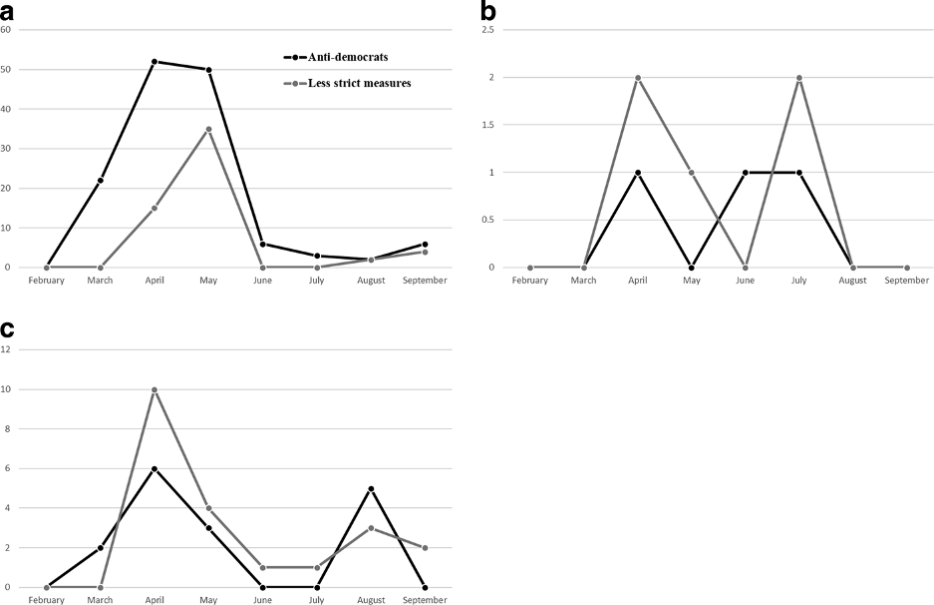
Anti-democratic accusations and demands for less measures over time. **a** Vox, **b** FPÖ, **c** AfD. (Absolute numbers of Tweets containing respective discourses per month)

The lack of nativist discursive opportunities has been compensated by the AfD, FPÖ and Vox by using a particular demonizing discourse against political elites accusing them of abolishing freedom and democracy and acting autocratic by maintaining pandemic related measures. Fig. 7 shows that in these three countries where such demonizing discourses are most salient, we see a correlation between these types of accusations and demands for less restrictions. For example, Vox accuses the Spanish government of taking advantage of the pandemic to impose repressive measures against the population. In this regard, they oppose extensions of the state of alarm in April and May (“the state of abuse”) considering it as an attempt to concentrate executive power, to violate the freedom of the citizens and to impose the governments’ “totalitarian agenda”.^7^ In this context, the party promoted public protests demanding the end of restrictions (“#faselibertad”) during May, while accusing the government of trying to censor these protests. In a similar vein, the AfD became the strongest opponents of pandemic-related measures among the German parties opposing face masks and restrictions in Germany—several politicians from the party further support the “Querdenker” movement openly spreading conspiracy theories (Utz et al. 2021). Demands for the end of restrictions are accompanied by demonizing accusations towards the government to abolish democracy in Germany. Regarding the Austrian FPÖ, the “anti-democratic” category hardly occurs, but if so mostly accompanied by demands for less restrictions. Thus, PRRP try to mobilize the unrest that the harsher measures of the first wave of the pandemic had caused in parts of the population to attack the political elites in *populist* terms—acting against the will of the people, trying to concentrate power and censoring the opposition.

## Discussion

The Covid-19 pandemic confronted populist radical right parties with a challenging discursive scenario since especially nativist discourses could be less demanded in times of an existential health crisis, at least temporarily. In this article we aimed to analyze how PRRP adapted discursively to this unprecedented scenario. We first showed that PRRP emphasize on rhetorical attacks towards domestic political elites and only sporadically accuse the media and foreign targets—it is mostly the European Union (and China in the case of Vox) being occasionally criticized as “externals”. Also scientists and academic elites—strongly present in the media during the pandemic—are hardly attacked. In sum, the pandemic is framed as a domestic political crisis in 2020 and not primarily as an international crisis threatening public health.

As expected, outgroups and immigration are not the main targets of PRRPs’ blame attribution during the pandemic. Mostly at the beginning of the pandemic PRRP attempted to link their initial support for restrictions to blame attributions towards supposed non-native outgroups—primarily immigrants and religious/cultural groups—accused of not following the restrictions and spreading the virus. Here, the authoritarian orientation of PRRP became evident, demanding that infringements of authority are to be punished severely (Mudde 2007). This pattern is in line with the findings of Hartman et al.’s (2021) study which establishes a positive relationship between the anxiety generated by the threat of the virus and the salience of authoritarian attitudes towards out-groups. As we could show, attacks on out-groups and nativist claims were indeed more frequent at the beginning of the pandemic, when the virus was considered a big threat. These nativist discourses overlap with messages calling for tougher measures against the spread of the virus and against those who did not comply with restrictions. Yet, almost all PRRP changed their discursive strategy becoming less supportive of measures to avoid the spread of the virus when the number of infections began to fall. As a consequence, blaming immigrants for not following restrictions or demands for border closure could not be sustained because they no longer fit the discursive context of the pandemic.

**Fig. 8:**
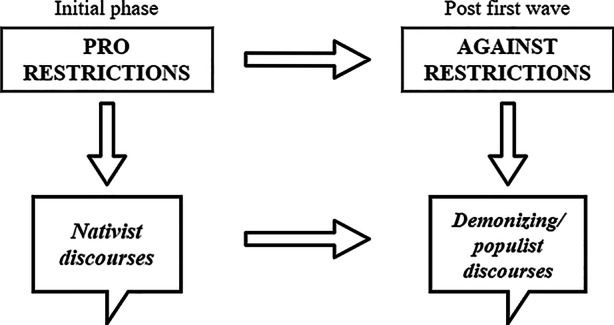
Development of PRRPs’ strategies and discourses in Spain, Austria and Germany

Three of the six populist parties—Vox, AfD and FPÖ—tried to compensate the lack of nativist blame attribution by focusing on demonizing populist rhetoric accusing the government of being “evil” by abolishing democracy and freedom through certain measures (Fig. 8). Thus, a *populist turn* occurred when numbers of deaths and infections seemed to fall and the actions of political elites were identified as the real threat by PRRP. The fact that the SVP—which generally attacked political elites less frequently than other PRRP—hardly uses demonizing accusations may be explained with its role as governmental party in the Swiss Federal Council. RN in France and Chega in Portugal did not experience the same kind of populist demonization turn as Vox, AfD and FPÖ either and used few demonizing statements only in the context of demands for stricter measures. While these parties were in opposition and not restricted by deliberation with coalition partners (Bernhard 2020), national peculiarities and different party agendas might explain the adoption of differentiated strategies at that stage of the pandemic. As argued by (Chazel 2020), for example, FN took the strategic decision to support pandemic related measures criticizing the government of being too permissive. Yet, its pro-restriction approach was compatible with conspiracy myths about the origin of the virus the party indirectly has spread (Chazel 2020). In this sense, becoming pandemic deniers is no inevitable deterministic development since demanding restrictions can be compatible with PRRPs’ ideological orientation as well. The fact that PRRP did not respond homogenously to the pandemic shows how far the pandemic issue is from the actual agenda of PRRP.

## Conclusion

Recapitulating, one might argue that it was the *populist* orientation of PRRP that saved them discursively and electorally during the pandemic and not their *nativism *either in order to blame the elite for not acting in time and being unprepared or for introducing authoritarian measures and undermining freedom and democracy. This raises the question whether nativism is indeed the dominant ideological feature of PRRP and whether their populism should rather be neglected as suggest by some scholars (Akkerman 2017; Rydgren 2017). Yet, we suggest to be cautious here. We admit that nativism may not be as indispensable to PRRP as it is commonly assumed. But that does not mean that populism is the main ideological feature of PRRP. Data from existing pre-pandemic studies suggest that nativist elements are at least as important for PRRP than populist ones—if not even more important (Schwörer 2021b). The pandemic is an extraordinary situation for PRRP and rather underlines the flexibility of PRRPs’ ideological toolkit, which can shift the emphasis from nativist to populist discourses and vice versa.

It is usually assumed that PRRP are less responsive to public attitudes or party system developments than mainstream parties (Adams et al. 2006; Fagerholm 2016). While we could not provide proof for any causal relationship, we observed that PRRP communicative strategies changed during the pandemic in the face of external developments. Thus, we should not consider PRRP as immune to public opinion or other external factors regarding positions on issues PRRP do not own. The first wave forced PRRP in all countries to discursively react to the pandemic and the different degree of affectedness seems to have influenced PRRP’s standpoints towards the pandemic and their messages against the government. Yet, speaking with Moffit (2015, p. 195), some PRRP tried to “actively perform and perpetuate a sense of crisis”—a different crisis than the actual pandemic, caused by anti-democratic malicious political elites and not predominantly by a potentially deadly virus—once the first wave abated.

The findings provide important implications for future research and political discussions. We should not expect that PRRPs’ ideology is too “thin” in order to connect to new party system agendas and discursive panoramas. On the contrary, this research shows how PRRP are able to electorally survive a pandemic that does not deliver favorable nativist discourses opportunities by emphasizing their populist profile and blaming elites without references to immigration. Especially the fact that PRRP did not experience substantial vote losses during the pandemic as often predicted (Wondreys and Mudde 2020) shows that they can “survive” periods with challenging opportunity structures due to a discursive flexibility provided by their populist ideology. This may also account for other crises and events that suddenly put new issues on the party system agenda that PRRP cannot escape such as Russia’s war in Ukraine or the climate crisis (which, admittedly is not such a “sudden” event but becomes much more salient in public debates recently). While PRRP may struggle finding their way to address new issues, their populist orientation is flexible enough to link them to their anti-elitist profile. Furthermore, crises are mostly considered as a *temporarily* worsening of a situation (Spier 2010) meaning that single issues also loose saliency as soon as shocks are publicly digested. In the long run, PRRP may get discursive opportunities to reemphasize their traditional topics.

## Supplementary Information

Appendix

## Footnotes

1 While the question if populist discourses derive from an ideology is not the main topic in this paper, we (the authors) belief that populist leaders share an aversion to elites, which provides them with a (limited) interpretive framework for political phenomena. In this sense, we rather agree with the ideational approach to populism.

2 “vorübergehende Zuspitzung der Lage”.

3 See collected data from Politico: https://www.politico.eu/europe-poll-of-polls/.

4 https://www.ecdc.europa.eu/en/covid-19/timeline-ecdc-response.

5 The share of Tweets containing a negative reference to political elites on the total amount of messages *containing a pandemic keyword* fluctuates between 32% (SVP) and 67% (Vox) and the share of such messages on Tweets containing accusations towards targets varies between 78% (AfD) and 97% (Chega).

6 The frequency of other allegations and types of blame attributions towards domestic political elites is illustrated in the appendix.

7 Example: “(…) The social communist government must listen to the clamor of the Spaniards asking for Freedom. I will remind it tomorrow in the tribune of the Congress, opposing to extend a dictatorship camouflaged as an alarm.” (Vox, 19/5/2020).
