# Supplementary material for: Populist radical right parties and discursive opportunities during Covid-19. Blame attribution in times of crisis
Source: Z Vgl Polit. 2022 Nov 21;16(3):545–70. doi: 10.1007/s12286-022-00540-w (PMC9684866; doi:10.1007/s12286-022-00540-w)
Supplement: Supplementary file 1 — Appendix [file 12286_2022_540_MOESM1_ESM.docx]

**Appendix**

**Populist radical right parties and discursive opportunities during Covid 19. Blame attribution in times of crisis**

*Jakob Schwörer and Belén Fernández-García*

**A1.** Dictionary with pandemic-related keywords 1

**A2.** Category system for empirical measurements 3

**A3.** Examples of non-pandemic related Tweets (not containing a keyword) 5

**A4**. Frequencies of different types of accusations/demands towards political elites 8

**A1.** Dictionary with pandemic-related keywords

| **German** | **French** | **Spanish** | **Portuguese** |
| --- | --- | --- | --- |
| Covid | Covid | Covid | Covid |
| Corona | Corona | Corona | Corona |
| Krise | Crise | Crisis | Crise |
| Virus | Virus | Virus | Virus |
| Sars | Sars | Sars | Sars |
| Epidemi | Épidémie | Epidemia | Epidemia |
| Pandemi | Pandémie | Pandemia | Pandemia |
| Maske | Masque | Mascarilla | Máscara |
| Mund-Nase |  |  |  |
| Gesundheit | Sanitaire | Sanitaria | Sanitária |
|  | Santé | Sanidad | Saúde |
|  |  | Salud |  |
| Notfall | Urgence | Alarma | Emergência |
| Ausnahmezustand |  |  |  |
| Tod | Mort | Muert | Mort |
| Tote |  |  |  |
| Verstorben |  |  |  |
| Sterblichkeit | Mortalité | Mortalidad | Mortalidade |
| Mortal |  |  |  |
| Normalität | (Nouvelle)Normalité | (Nueva)Normalidad | (Novo)Normal |
| Impf | Vaccin | Vacuna | Vacina |
| Quarantäne | Quarantaine | Cuarentena | Quarentena |
|  | Confin | Confina | Confina |
| Lockdown | Lockdown | Lockdown | Lockdown |
| PCR | PCR | PCR | PCR |
| Infekt | Infect | Infec | Infec |
| Infiziert |  |  | Infetado |
| Infektiös | Contagion | Contagi | Contágio |
| Ansteck |  |  |  |

**A2.** Category system for empirical measurements

| Category | Subcategory | Operationalisation | Example sentences |
| --- | --- | --- | --- |
| Messages against the political elite | General accusation of bad management without clear tendency | General accusation of bad crisis management without concrete demands in favour or against restrictions  *Related to measures and pandemic management (not to economic/social consequences)* | “Since the beginning of this health crisis, a certain number of strategic choices seem to me to be questionable. These choices have largely undermined the confidence of the French in this government, and I understand them.” (RN 25/3/2020) |
|  | Manipulation, spreading lies and fake news | Lie; hide information; manipulate; spread misinformation and panic; are selfish; corrupt; interested in own carreer or in their own political agenda  *Related to measures and pandemic management (not to economic/social consequences)* | “Cleaning up the #Corona #lies + + + The #mask is the symbol of government obedience!” (AfD 13/9/2020) |
|  | Anti-democratic attitude | Are antidemocratic; totalitarian; act against the constitution or against the will or well-being of the people; demonize, ridicule, exclude political opponents and critics of their crisis management and measures; want to surveil society  *Related to measures and pandemic management (not to economic/social consequences* | “@Macarena_Olona ‘This #BigBrotherGovernment is using the Coronavirus crisis as an alibi to impose repressive measures.’ @VOX_Congreso requests the creation of an investigative subcommittee after Marlaska acknowledged that they monitor social networks. (VOX 22/4/2020) |
|  | Stricter/earlier measures to fight the pandemic | Demands for stronger measures against the virus; elites do not take the crisis seriously; have no plan against crisis; have contributed to the spread of the virus  *Related to measures and pandemic management (not to economic/social consequences)* | “How long will this unconsciousness rule us? QUARANTINE mandatory and closing BORDERS NOW! #CHEGA” (Chega 16/3/2020). |
|  | Less strict measures; less/no further restrictions | Should revoke measures; should not take further measures; elites take the crisis to serious or overrate it  *Related to measures and pandemic management (not to economic/social consequences)* | “Voluntary wearing of masks in public spaces instead of state-imposed mandatory wearing of masks. The AfD parliamentary group in Bavaria demands the rapid relaxation of the restrictions on daily and economic life that apply to the Free State.” (AfD 21/4/2020) |
|  | Nationalist and nativist demands and accusations | Demanding nationalist policies including measures and accusations against non-natives and in favour of border control and closure; demanding less solidarity with other countries and a strong nation state  *Related to measures but also to economic/social aspects of the pandemic management* | “Restricting freedom of movement should have been an obvious precaution, yet the government has provided an ideological response by refusing any border control!” (RN 6/3/2020) |
|  | Worsening and producing economic/social problems | Not providing sufficient help for economic sectors, vulnerable groups etc.; waste public resources (or resources that should be allocated to the needs of the people); demands for not harming the economy  *Only related to economic/social consequences and not to the pandemic managements* | “We want the return to real normality and have nothing to do with the new normality of @sebastiankurz! The measures of the government have cost countless jobs and livelihoods!” (FPÖ 29/4/2020) |
|  | Non-pandemic related negative evaluations/ demands of all kind | Elites are evil, anti-democratic, manipulate etc.  *Not related to the topic of the pandemic and its social/economic consequences* | “After #shootings and #forced labour for #rich the next proposal of the ex-#SED: Left faction leader #Bartsch demands a ""#Corona levy"" on large private fortunes.” (AfD 26/3/2020) |
| Messages against non-native outgroups | Don't respect restrictions; contribute to the spread | Immigrants, Muslims, other ethnic or religious minorities, etc. are spreading the Virus, do not accept the restrictions; emphasizing that non-natives are often infected with the virus  *Related to measures and to economic/social aspects of the pandemic management* | “Illegal immigrant riots, worsening #COVID19 epidemic in #Mayotte... the state must react urgently! Send troops to restore order and bring back a hospital ship to prevent the situation from getting out of hand!” (RN 4/5/2020)  “The invasion of the Monforte Barracks is proof that, in the fight against the covid-19, the Roma community does not speak the same language as the majority. This distance from common values, this almost parallel state, cannot remain this way. Courage continues to lack!” (Chega 21/4/2020) |
|  | Preferentially treated | Immigrants, Muslims etc. are portrayed as being preferentially treated and of not having to comply certain restrictions  *Related to measures and to economic/social aspects of the pandemic management* | “Instead of fulfilling the constitutional mandate and protecting the people in Switzerland from criminal foreigners, they are simply set free in the Corona crisis and can go into hiding with the approval of the judiciary and the authorities.” (SVP 23/4/2020) |
|  | Non-pandemic related negative evaluations/ demands of all kind | Any other negative evaluation and demands towards outgroups not related to the topic of the pandemic  *Not related to the topic of the pandemic and its social/economic consequences* | “During the epidemic, all of Spain was paralyzed, except for the importation of illegal immigrants. The government not only fails to curb the organized invasion, but also promotes the call effect. Let's stop the INVASION!” (VOX 14/9/2021) |
| Anti-immigration messages  Pro national sovereignty | Anti-immigration | Mentions of critical stances towards immigration with or without concrete references to outgroups; demands for expulsions | “They want a Europe with cheap labour and subjugated Europeans without families. Illegal immigrants who disembarked this week infected by Covid, for being young, will be entitled to respirators that have been and may be lacking in the future to our elders.” (VOX 19/6/2020). |
|  | Pro sovereignty | Explicit references for the protection and closure of borders (also pandemic-related); protection of national sovereignty and national products/industries  *This category mostly refers to pandemic-related claims for border control and economic and national sovereignty* | “We are only prepared for the next crisis if we can provide ourselves with these vital goods” (SVP 29/4/2020)  “The enemies of the sovereignty of nations want to take advantage of this crisis to accelerate their globalist agenda. Pedro Sánchez, puppet of the globalists and friend of Soros, has confirmed this in Congress. VOX will stand up to them. Spain will prevail.” (VOX 22/4/2020) |

**A3.** Examples of non-pandemic related Tweets (not containing a keyword)

| **Party** | **Example** |
| --- | --- |
| AfD | (20.9.) Obama war geschmeidig und elegant – und brachte Krieg.  (18.8.) Vor einem Jahr feierte sich die @cducsubt für ihr Gesetz zum Passentzug für #Gefährder. Umgesetzt wurde es seitdem genau Null mal.  (3.7.) Die Bundesregierung macht mutwillig entscheidende Wirtschaftszweige zunichte, ohne vorher Ersatzkonzepte zu Ende gedacht zu haben. Die #AfD bleibt bei ihrem NEIN zum überhasteten #Kohleausstieg!  (30.6.) Unser Land muss wieder lebenswert und sicher werden!  (21.5.) Mit wievielen Kurzarbeitern rechnet die #Bundesregierung (~2.500.000),wie teuer wird das (#Kurzarbeitergeld)?  (24.4.) ++ Jeder zweite "#Flüchtling" klagt - #Linke: Es sollen noch mehr werden! ++ #BAMF soll „ablehnende Bescheide vermehrt eigenständig überprüfen und sie gegebenenfalls zugunsten der #Asylsuchenden korrigieren“.  (20.3.) #Seehofer verbietet erstmals eine #Reichsbürger-Gruppierung bundesweit. @Beatrix_vStorch: „Ich begrüße die Verbotsentscheidung und fordere den Innenminister auf, konsequent zu bleiben und die linksextremistische Internetplattform #Indymedia zu verbieten!  (27.2.) ++ Unglaublich: Wie arabische und osteuropäische #Großfamilien gezielt unsere #HARTZIV-Kassen plündern! ++ Ein Datenleck einer Arbeitsagentur liefert erschreckende Einblicke in den organisierten #Betrug von ausländischen #Clans. |
| FPÖ | (9.9.) Kreislaufwirtschaft bei der #ÖVP: Eine Mitarbeiterin aus Sobotkas Präsidentenbüro wirbt nebenberuflich Inserate für das Mock-Institut, #Novomatic schaltet dort Inserate - und das Mock-Institut inseriert in der Zeitung des NÖAAB. Geld in der Parteikasse …  (20.8.) Wer ist wohl dieser ehemals im Innenministerium beschäftigte ÖVP-nahe Berater, der den Kontakt zu Marsalek hergestellt hat?  (17.7.) Wäre schon spannend zu wissen, wer aller die Adressaten der ÖVP-USB-Sticks waren...dürfte inhaltlich auch eher selektiv gewesen sein  (24.6.) Kanzler @sebastiankurz sagt, er weiß nicht, wer hinter dem Ibiza-Video steckt. Dafür hat er aber gegenüber Strache und auch öffentlich ganz schön spekuliert  (14.5.) Kickl: Kanzler Kurz führt im Klein Walsertal seine eigenen Regeln ad absurdum  (18.4.) Österreich wird zum Bespitzelungs-, Denunzianten- und Überwachungsstaat. Es ist eine Schande!  (4.3.) Die illegalen Migranten befinden sich auf dem Weg nach Europa! Die einzig richtige Botschaft lautet: NO WAY Dass Schwarz-Grün in dieser Situation versagt, ist offensichtlich. Herbert Kickl fordert ein Grenzübertrittsverhinderungspaket  (20.2.) Aufgedeckt: 72 Jihad Rückkehrer derzeit in Österreich... |
| SVP | (17.9.) Nationalrat und Unternehmer Thomas Matter räumt mit dem Märchen auf, Schweizer Unternehmen verlören bei einem Ja zur Begrenzungs-Initiative den Zugang zum EU-Markt oder könnten keine Arbeitskräfte mehr im Ausland rekrutieren. Das Gegenteil ist wahr.  (21.8.) Wir wollen doch nicht die Schweizer Löhne auf den Minimallohn senken. Ja zur #Begrenzungsinitiative  nativism stop immigration because harmful  (15.7.) Die Schweiz darf sich der EU nicht unterwerfen, sondern muss die Zuwanderung wieder selber steuern! JA zur #Begrenzungsinitiative am 27. September!  (23.6.) Die Schweiz soll ein Land voller Rassisten sein, nur weil sie zu einer Süssigkeit Mohrenkopf sagen? Offensichtlich stimmt das nicht - sonst würden sich wohl kaum jedes Jahr Zehntausende Ausländer in der Schweiz niederlassen.  (18.5.) Die SVP verlangt griffige Polizeiliche Massnahmen zur Terrorbekämpfung.  (28.4.) Die SVP fordert, dass jetzt zuerst den Menschen in der Schweiz geholfen werden muss. Der Import von Wirtschafts- und Sozialmigranten aus aller Welt ist sofort zu stoppen.  (18.3.) Der Bundesrat hat heute entschieden, die Volksabstimmung vom 17. Mai nicht durchzuführen. Die SVP Schweiz nimmt dies zur Kenntnis und steht hinter dem bundesrätlichen Entscheid.  (26.2.) Studie bestätigt: Arbeitslose über Fünfzig bekommen keinen Job. Darum Ja zur Begrenzungsinitiative! #BGIja #eswirdeng #Abst20 #CHVote |
| Vox | (30.9) Esto tiene que terminar. Los españoles decentes no pueden aguantar ni un minuto más en sus barrios las agresiones, los robos o las violaciones de los menas. En VOX lo tenemos claro: todos los menas han de ser detenidos y expulsados de vuelta a África.  (31.8) Que no tengan ninguna duda los golpistas, defenderemos la unidad de España hasta las últimas consecuencias #EspañaSiempre  (31.7) La Universidad, los colegios; la educación en general está secuestrada por el separatismo totalitario. Entregaron las competencias y lo han aprovechado para inocular odio, generar confrontación y manipular la historia de España.  (15.6) A las 21.15 en @EstadoDAlarmaTV con @InesCanizares de @vox_es @CristinaSegui_ y @MargaProhens de @populares q saca de quicio a @IreneMontero. Hablaremos de su última ridiculez feminazi, de las leyes de género, de ZP o del control antidroga d @Santi_ABASCAL  (31.5) A las 23h llega @hermanntertsch de @vox_es y @benjalh1971 con su Eje del mal y las prácticas chavistas de Pablo Iglesias, el hijo del FRAP. Siga el estreno aquí https://t.co/bM7lwhLdSr  (30.4) Hoy con @Ortega_Smith de reparto por el Distrito de Tetuán. https://t.co/WyhULzXCvZ  (31.3) Santiago Abascal presenta el programa: Protejamos España https://t.co/HkkZEVDMVu vía @YouTube |
| Chega | (28.9) Mentira !15 milhões dos nossos impostos ... https://t.co/U7D56OpACO  (31.8) O medo que o sistema tem é tanto, tanto, que só podemos estar no caminho certo! André Ventura.  (30.7) Domingo às 17:30 na Praça do Município, em Lisboa! Contamos consigo!  (30.6) Ah essa nazi do Bloco de Esquerda. Tenham vergonha!!! Deixem de ser ridículos!  (31.5) A notícia que os bloquistas e os comunistas não queriam ter este domingo,mas tem de ser. Obrigado Portugal . Até à vitoria!  (30.4) Chegou a altura de uma nova política de habitação. Em vez de compadrios com os grandes investidores e fundos de investimento o que as nossas cidades precisam é de uma política de habilitação acessivel, justa, e que não discrimine a classe média e os portugueses que não são ricos.  (24.3) Governo está mais preocupado com os presos que com os guardas prisionais!  (27.2) CHEGA desmascara PS sobre comissões bancárias! via @YouTube |
| RN | (30.9) En raison de leurs convictions religieuses, certains individus agressent des femmes au motif qu'elles portent des jupes. C'est un vrai problème et nous attendons de la part du gouvernement des solutions sur le sujet du séparatisme !  (31.8) À noter dans vos agendas! Rentrée politique du Rassemblement National ce dimanche à #Fréjus: le discours de Marine Le Pen sera diffusé en direct, à partir de 15h, sur YouTube, Facebook et Twitter  (31.7) #AïdElKebir: le gouvernement français doit arrêter la ségrégation halal !#Halal #EidAladha #CauseAnimale Retrouvez mon communiqué  (30.6) Au-delà de nos vifs et profonds désaccords politiques, ce type de procédure sent l’acharnement à plein nez. Abuser de son pouvoir contre l’opposition quelle qu’elle soit est un aveu de faiblesse et suscite l’inquiétude sur notre État de droit et notre fonctionnement démocratique.  (30.5) Nouvelle offensive contre le Parlement européen à #Strasbourg! Derrière ces attaques, c'est la France et les Français qui sont visés. Envoyez un message clair à la Commission européenne et à Macron, signez notre pétition.  (30.4) Les affronts subis avec flegme et dignité par nos policiers sont d’effroyables humiliations pour la République qui s’incline et recule devant la voyoucratie et la tyrannie des racailles ! Retrouvez ma question écrite à @CCastaner  (27.3) Les bonnes nouvelles sont rares, celle-ci en est une. Bon retour à nos trois compatriotes dont la disparition avait vivement inquiété de nombreux Français. MLP https://t.co/lehAWv7Nb5  (29.2) «Emmanuel Macron est un Président autoritaire, particulièrement isolé. Nous le disons aux Français : le seul moyen désormais de remettre en cause cette #RéformeDesRetraites, c'est de voter @MLP_officiel en 2022 ! » #49al3 @BFMTV |

**A4.** Frequencies of different types of accusations/demands towards political elites

Note: Standard deviations in brackets.
